# Machine learning prediction of dropping out of outpatients with alcohol use disorders

**DOI:** 10.1371/journal.pone.0255626

**Published:** 2021-08-02

**Authors:** So Jin Park, Sun Jung Lee, HyungMin Kim, Jae Kwon Kim, Ji-Won Chun, Soo-Jung Lee, Hae Kook Lee, Dai Jin Kim, In Young Choi

**Affiliations:** 1 Department of Medical Informatics, College of Medicine, The Catholic University of Korea, Seoul, South Korea; 2 Department of Biomedicine & Health Sciences, College of Medicine, College of Medicine, The Catholic University of Korea, Seoul, South Korea; 3 Department of Psychiatry, College of Medicine, The Catholic University of Korea, Seoul, Korea; 4 Department of Psychiatry, Seoul St. Mary’s Hospital, College of Medicine, The Catholic University of Korea, Seoul, South Korea; Taipei Medical University, TAIWAN

## Abstract

**Background:**

Alcohol use disorder (AUD) is a chronic disease with a higher recurrence rate than that of other mental illnesses. Moreover, it requires continuous outpatient treatment for the patient to maintain abstinence. However, with a low probability of these patients to continue outpatient treatment, predicting and managing patients who might discontinue treatment becomes necessary. Accordingly, we developed a machine learning (ML) algorithm to predict which the risk of patients dropping out of outpatient treatment schemes.

**Methods:**

A total of 839 patients were selected out of 2,206 patients admitted for AUD in three hospitals under the Catholic Central Medical Center in Korea. We implemented six ML models—logistic regression, support vector machine, k-nearest neighbor, random forest, neural network, and AdaBoost—and compared the prediction performances thereof.

**Results:**

Among the six models, AdaBoost was selected as the final model for recommended use owing to its area under the receiver operating characteristic curve (AUROC) of 0.72. The four variables affecting the prediction based on feature importance were the length of hospitalization, age, residential area, and diabetes.

**Conclusion:**

An ML algorithm was developed herein to predict the risk of patients with AUD in Korea discontinuing outpatient treatment. By testing and validating various machine learning models, we determined the best performing model, AdaBoost, as the final model for recommended use. Using this model, clinicians can manage patients with high risks of discontinuing treatment and establish patient-specific treatment strategies. Therefore, our model can potentially enable patients with AUD to successfully complete their treatments by identifying them before they can drop out.

## Introduction

According to a 2016 Korean epidemiological survey on mental illness, the lifetime prevalence of alcohol use disorders (AUDs), including alcohol dependence and abuse, was 12.2% (18.1% for men and 6.4% for women), which is the highest among mental disorders [1]. AUDs result in significant economic losses, various social problems such as alcohol-related crimes and accidents, and physical diseases such as alcohol-induced physical complications and alcohol-related dementia [2–4].

AUD is a disease with a higher recurrence rate than that of other mental illnesses [5–7]. To prevent recurrence, the disorder must be managed over a long time without stopping the treatment at all [8, 9]. Moreover, steady treatment can positively influence the treatment outcome, such as prevention of recurrence [10–13]. In other words, continuous follow-up from the patient is an important indicator of prognosis [14].

However, the rate of outpatient treatment duration in patients with AUD is significantly low. According to a domestic study, 91.7% of patients stopped follow-up within six months of discharge [14]. In other countries, 52–75% of patients receiving outpatient treatment for alcohol abuse and dependence discontinued the treatment upon the fourth installment [15–17]. Therefore, predicting and managing patients with AUDs likely to drop out of follow-up is of paramount importance.

With the aim of increasing the retention rate of treatments in patients with AUDs, factors affecting the continuous maintenance of outpatient care have been studied. It was found that age, sex, physical and mental comorbidities, hospitalization, family history, type of drugs, marital status, drinking volume, and drinking period were factors that affected continuous outpatient visits [18–20]. However, these studies were mostly prospective, and retrospective studies focused only on factor analysis, using traditional methodologies such as logistic regression [14].

In recent psychiatric research, machine learning (ML) models have been used to predict psychiatric disorders with high accuracy, which is useful for developing clinical decision support systems and identifying influential variables [21–24]. In the United States, a study predicted success in treatment of patients with substance use disorders. However, this study was not very meaningful as it compared the performance of various ML models rather than identifying factors [25]. In this regard, a study was conducted to predict the discontinuation of inpatient treatment for opioid abuse patients using the Treatment Episode Data Set—Discharges claim data from Substance Abuse and Mental Health Services Administration in the United States [26]. However, in psychiatry, success criteria for inpatient and outpatient treatment are defined differently, depending on the duration of treatment [25]. In other words, predicting treatment discontinuation in outpatients with AUD is challenging.

Currently, no studies that predict treatment dropout rates in outpatients with AUDs in Korea have been published. Therefore, this study aimed to predict the discontinuation of outpatient treatment in patients with AUD via ML. The obtained model can aid personalized patient management so that patients with AUDs can maintain treatment steadily. Ultimately, the model could prevent recurrence and increase the success rate of treatments for such patients.

## Materials and methods

### Experimental data

**Fig 1:**
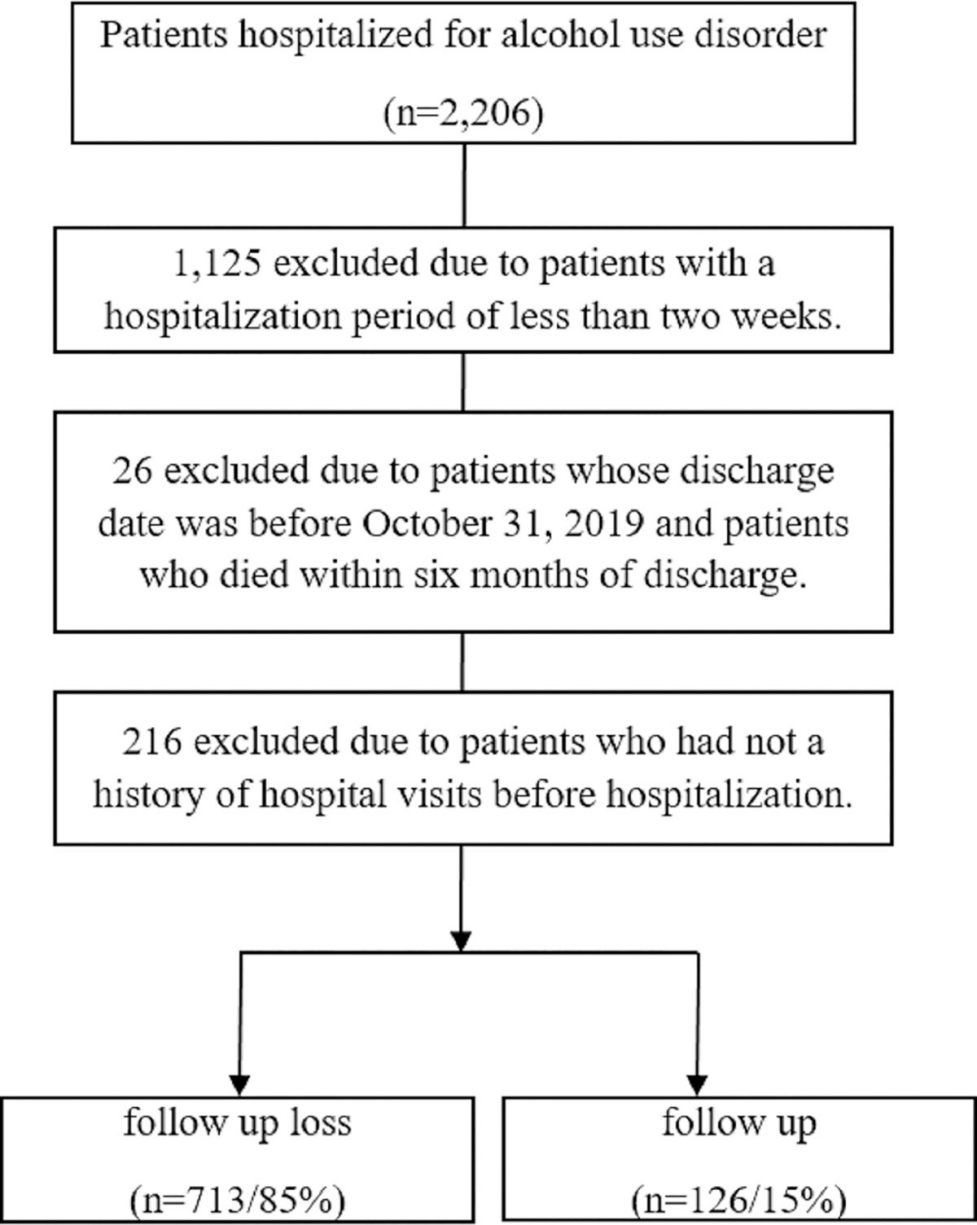
Flow chart of inclusion of subjects.

This was a multicenter retrospective study of patients visiting Seoul St. Mary’s Hospital, Uijeongbu St. Mary’s Hospital, and Bucheon St. Mary’s Hospital in Korea. The study considered data from 2,206 patients, hospitalized between January 2006 and March 2020, for mental and behavioral disorders due to alcohol use (F10, ICD-10). The study protocol was approved by the institutional review board of the Catholic University of Korea (IRB No. XC20WIDI0079K). Data were collected through the Clinical Data Warehouse (CDW), which incorporates eight affiliated hospitals under the Catholic Medical Center (CMC) in Korea. The CDW is a database that has completely anonymized approximately 15 million electronic medical records, and can extract data based on research characteristics. Thus, this study proceeded with consent exemption. The selection of the participants follows the process shown in Fig 1.

For this study, patients with AUD were defined as those with a hospitalization period of more than two weeks. Thus, the study focused on patients requiring continuous outpatient treatment. If more than two hospitalizations had occurred for more than two weeks, the first hospitalization was defined as the date of hospitalization. Follow-up success in patients with AUD was defined as outpatient visits at least once a month, for six months after discharge [14]. Therefore, patients with discharge dates before October 31, 2019 were excluded. Patients who died within six months of discharge were excluded, and only those who had a history of hospital visits before hospitalization were included to determine whether they had comorbidities. Consequently, the final dataset comprised 839 patients.

### Variable selection

In this study, we selected 11 variables based on prior research [16–18] and consultation with clinicians. Furthermore, we computed the variance inflation factor and constructed the variables without multicollinearity between them. The final variables determined were age, sex, length of hospitalization, address, medical department, comorbidities diagnosed within a year before hospitalization (diabetes, liver disease, depressive disorder, and anxiety disorder), outpatient treatment for AUD before hospitalization, and prescription of naltrexone.

### Statistical analysis

**Fig 2:**
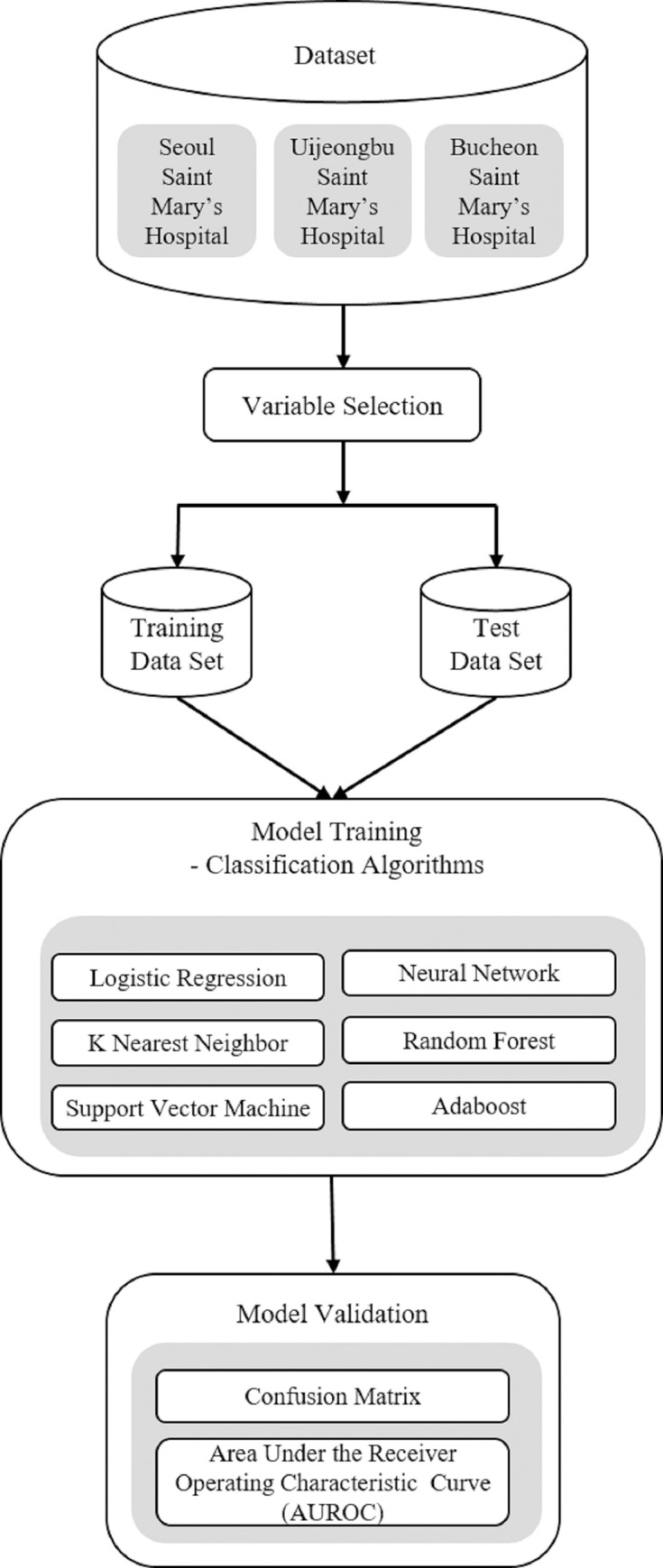
Block diagram of the process of the research analysis.

The data set of 839 patients was divided into ‘follow-up’ and ‘follow-up loss’ groups, depending on the duration of outpatient treatment and the number of outpatient visits. The follow-up group consists of patients who visited outpatient care for more than six months and more than once a month. In all other cases, it was classified as a follow-up loss group. As a result, the follow-up group and follow-up loss group consisted of 126 (15%) and 713 (85%) patients respectively. We performed chi-square tests for 11 categorical variables to determine the differences between the groups(p-value < 0.05, chisq.test in R). To evaluate the model, we split the data into 2 datasets: 80% for training and 20% for testing [27]. The collected data had a class imbalance problem of 85:15. The data imbalance problem was solved before training the model. When the ML model was applied to a highly imbalanced dataset, most learners exhibited a bias towards the majority classes, while ignoring minority classes [28]. Therefore, we applied oversampling to the training set to balance the classes [29]. Subsequently, we applied the ML model using 11 predictors from the training set. The ML models used were logistic regression [30], support vector machine [31], k- nearest neighbor (KNN) [32], random forest [33], neural network [34], and AdaBoost decision tree [35]. The evaluation of the models considered accuracy, sensitivity, specificity, and area under the receiver operating characteristic curve(AUROC). The development of a machine learning model follows the process shown in Fig 2. The ML analysis was implemented in Python (version 3.8), and the analysis package Scikit-learn was used [36]. The ML algorithms used in the analysis are described below.

#### Logistic regression

Logistic regression is a model of association between a dependent variable and independent variables when the dependent variable is binary [37]. Logistic regression coefficients can be easily interpreted as indicators of variable importance [38]. Therefore, it is a widely used model in healthcare. Furthermore, logistic regression can improve performance by considering regularization (L1, L2), to prevent overfitting. However, logistic regression has limitations in terms of solving nonlinear problems because linearity between dependent and independent variables must be assumed. For this study, the LogisticRegression algorithm in Scikit-Learn is used to model the dataset and l2 regularization specified.

#### Support vector machine

SVM is available for both classification and regression analysis, and is a supervised learning model for pattern recognition and data analysis in ML. SVM is a method of computing hyperplanes that optimally separate data belonging to two classes [39]. In addition to linear classification, SVM also enables nonlinear classification using kernel tricks. However, for large-scale data, the training time is long and it is difficult to understand the individual effects in the final model. This study used the linearSVC in Scikit-Learn.

#### K Nearest Neighbor

KNN is an algorithm that predicts output variables by referring to the nearest *k* neighboring points to a particular point. Both classification and regression models are possible. KNN can easily reflect newly accumulated data because it goes through a reasoning process without a learning process. However, the more the data and the larger the dimension, the more the increase in time and cost increase. In this work, we used the KNeighborsClassifier in Scikit-Learn with five neighbors using Euclidian distances. The Euclidean distance formula is shown below.


$$D(X,Y)=\sqrt{\sum_{i=1}^{n}{\left(x_{i}-y_{i}\right)}^{2}}\;where\;X=(x_{1},x_{2},\cdots ,x_{n})\;and\;Y=(y_{1},y_{2},\cdots ,y_{n}).$$

#### Random forest

Random forest is an ensemble method for learning multiple decision trees. It uses randomization techniques such as bagging to reduce the variance of performance. In addition, it solves the problem of overfitting, generating good prediction results. However, a large number of trees are required for accurate prediction, which can make the algorithm very slow and inefficient. Also, it is impossible to explain the associations between variables [40]. For this study, RandomForestClassifier in scikit-learn is used and gini impurity to measure the quality of a split.

#### Neural network

Neural networks are methods for predicting the values of target variables after learning, using numerous interconnected nodes within each layer consisting of input layers, hidden layers, and output layers. Recently, it has been a popular model in various fields due to its good predictive performance. Neural networks are prone to problems such as overfitting and underfitting due to their limited hidden layers and the complexity of learning. Furthermore, the results derived are difficult to explain.

#### AdaBoost

This is the first practical boosting algorithm studied by Freund and Schapire in 1997. This method leads to several weak learners and shows high performance because it creates a new predictor by relatively increasing the weight of poorly classified training samples based on errors from previous learners. Furthermore, the method has the advantage of being a tree-based algorithm, from which we can obtain the importance of variables that affect prediction, making it interpretable. This study used AdaBoostClassifier in scikit-learn.

## Results

We divided patients with AUDs as those who dropped out of outpatient treatment after discharge and those who received continuous treatment. A basic statistical analysis was conducted to determine which patient characteristics differed between the groups. Sex, address, medical department, depressive disorder, outpatient treatment for AUD before hospitalization, and prescription of naltrexone showed significant differences between the two groups (Table 1).

**Table 1:** Patient characteristics.

	Follow-up	Follow-up loss (n = 713)	P-value
	(n = 126)		
**Length of hospitalization**			0.406
Under 28d	437 (61.3%)	77 (61.1%)	
29-56d	136 (19.1%)	25 (19.8%)	
57-70d	110 (15.4%)	15 (11.9%)	
Over 70d	30 (4.2%)	9 (7.1%)	
**Sex**			0.008*
Male	91 (72.2%)	590 (82.7%)	
Female	35 (27.8%)	123 (17.3%)	
**Age**			0.058
Under 29	9 (7.1%)	22 (3.1%)	
30–39	22 (17.5%)	96 (13.5%)	
40–49	29 (23.0%)	201 (28.2%)	
50–59	30 (23.8%)	216 (30.3%)	
60+	36 (28.6%)	178 (25.0%)	
**Address**			0.04*
Seoul	37 (29.4%)	144 (20.2%)	
Gyeonggi	75 (59.5%)	451 (63.3%)	
Other	14 (11.1%)	118 (16.5%)	
**Medical department**			0.01*
Psychiatry	111 (88.1%)	546 (76.6%)	
Gastroenterology	9 (7.1%)	104 (14.6%)	
Other	6 (4.8%)	63 (8.8%)	
**Outpatient treatment for alcohol use disorder before hospitalization**			0.000*
No	35 (27.8%)	325 (45.6%)	
Yes	91 (72.2%)	388 (54.4%)	
**Diabetes**			0.087
No	109 (86.5%)	654 (91.7%)	
Yes	17 (13.5%)	59 (8.3%)	
**Liver disease**			0.224
No	107 (84.9%)	569 (79.8%)	
Yes	19 (15.1%)	144 (20.2%)	
**Depressive disorder**			0.006*
No	78 (61.9%)	529 (74.2%)	
Yes	48 (38.1%)	184 (25.8%)	
**Anxiety disorder**			0.053
No	104 (82.5%)	635 (89.1%)	
Yes	22 (17.5%)	78 (10.9%)	
**Naltrexone**			0.000*
No	93 (73.8%)	626 (87.8%)	
Yes	33 (26.2%)	87 (12.2%)	

* p<0.05

According to the results of the follow-up loss group, 82.7% of men and 74.2% of patients were found to not have depression. In addition, 87.8% of patients were not prescribed naltrexone and 63.3% of them were in the Gyeonggi Province. Lastly, 54.4% of patients opted for outpatient treatment for alcohol use disorder before hospitalization.

Table 2 shows the performance results of the machine-learning models analyzed using the 11 predictors. To compare the performance of the six models, we obtained the accuracy, specificity, sensitivity, and AUROC values. In this study, we selected the final model based on the AUROC value, considering both sensitivity and specificity, owing to the high class imbalance present in the data. Moreover, a grid search was performed with five-fold cross-validation to find the optimal hyperparameters of the ML models.

**Table 2:** The performance of machine learning algorithms.

Model	AUROC	Accuracy	Sensitivity	Specificity
Logistic Regression	0.6914	0.6130	0.7058	0.6026
SVM	0.6797	0.7023	0.6470	0.7086
KNN	0.6166	0.6726	0.5294	0.6887
Random Forest	0.6365	0.7380	0.4705	0.7682
Neural Network	0.6891	0.7440	0.5294	0.7682
AdaBoost	0.7241	0.6428	0.7647	0.6291

**Fig 3:**
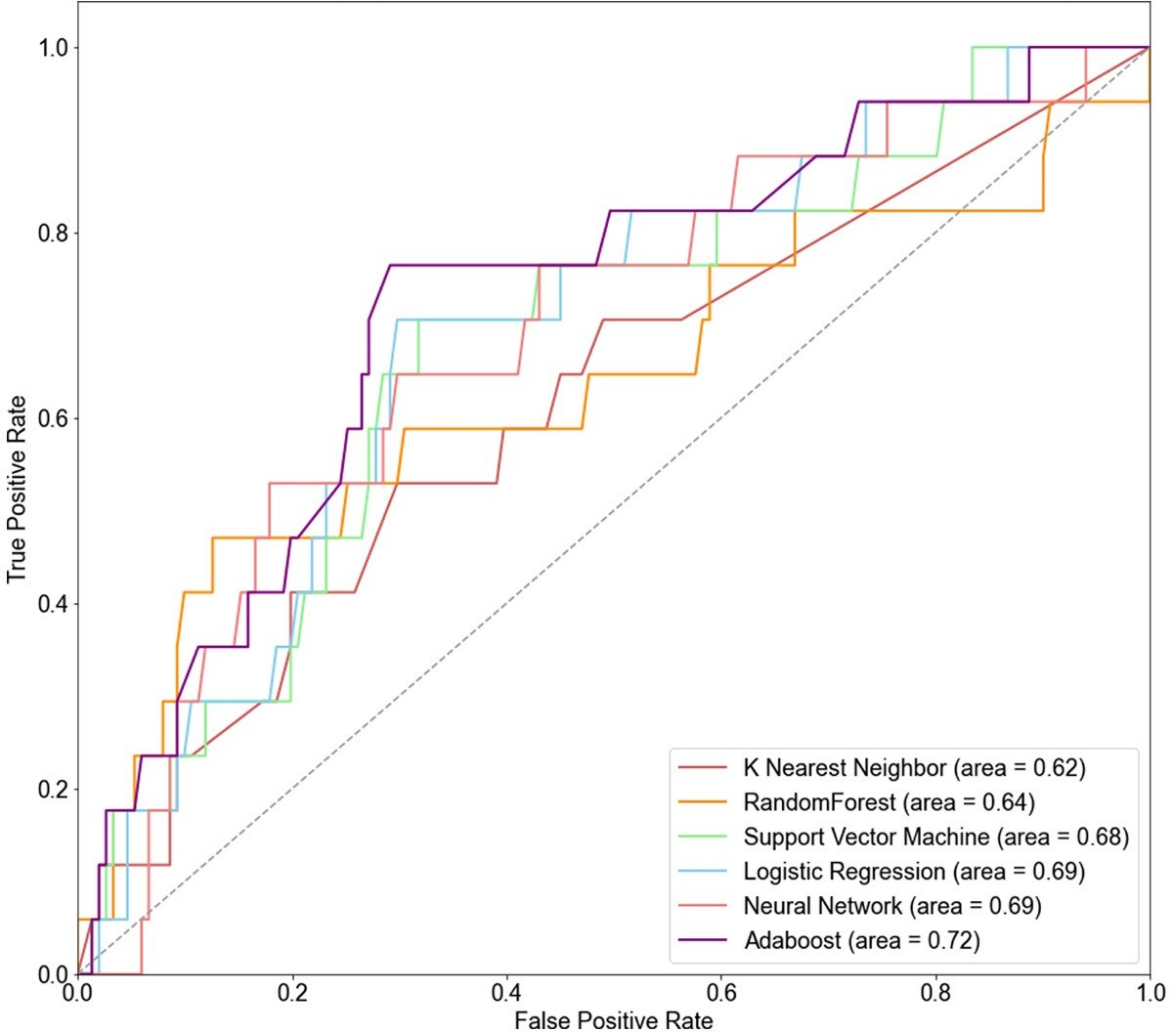
ROC curves of six different machine learning models.

Fig 3 shows the receiver operating characteristic (ROC) curves for the six models. The lower area of the curve drawn with each point is the AUROC value. The results show that the larger the area, greater is the AUROC value, and the higher is the performance. The results of the ROC curves show that AdaBoost has the highest AUROC of 0.72.

Table 3 shows the comparison of sampling methods to address data imbalance. First, we compared oversampling and undersampling. Upon comparing AdaBoost performance with random oversampling and random undersampling, the accuracy with of oversampling was higher by 0.065. Therefore, oversampling was considered as the preferred method. However, there is a possibility of overfitting because random oversampling is a method of randomly replicating data from the minority class. Next, we performed a comparison with other oversampling methods. The Synthetic Minority Oversampling Technique (SMOTE) method does not simply replicates minority data but uses the KNN algorithm to generate synthetic data. However, SMOTE does not take into consideration neighboring examples can be from other classes. This can increase the overlapping of classes and can introduce additional noise. After application of the SMOTE, performance was low. In other words, random oversampling showed the best results for our data.

**Table 3:** Comparison of imbalanced data set sampling methods.

Method	AUROC	Accuracy	Sensitivity	Specificity
Random Undersampling	0.6505	0.5773	0.7058	0.5629
Random Oversampling	0.7241	0.6428	0.7647	0.6291
SMOTE	0.6427	0.5952	0.5294	0.6026

**Fig 4:**
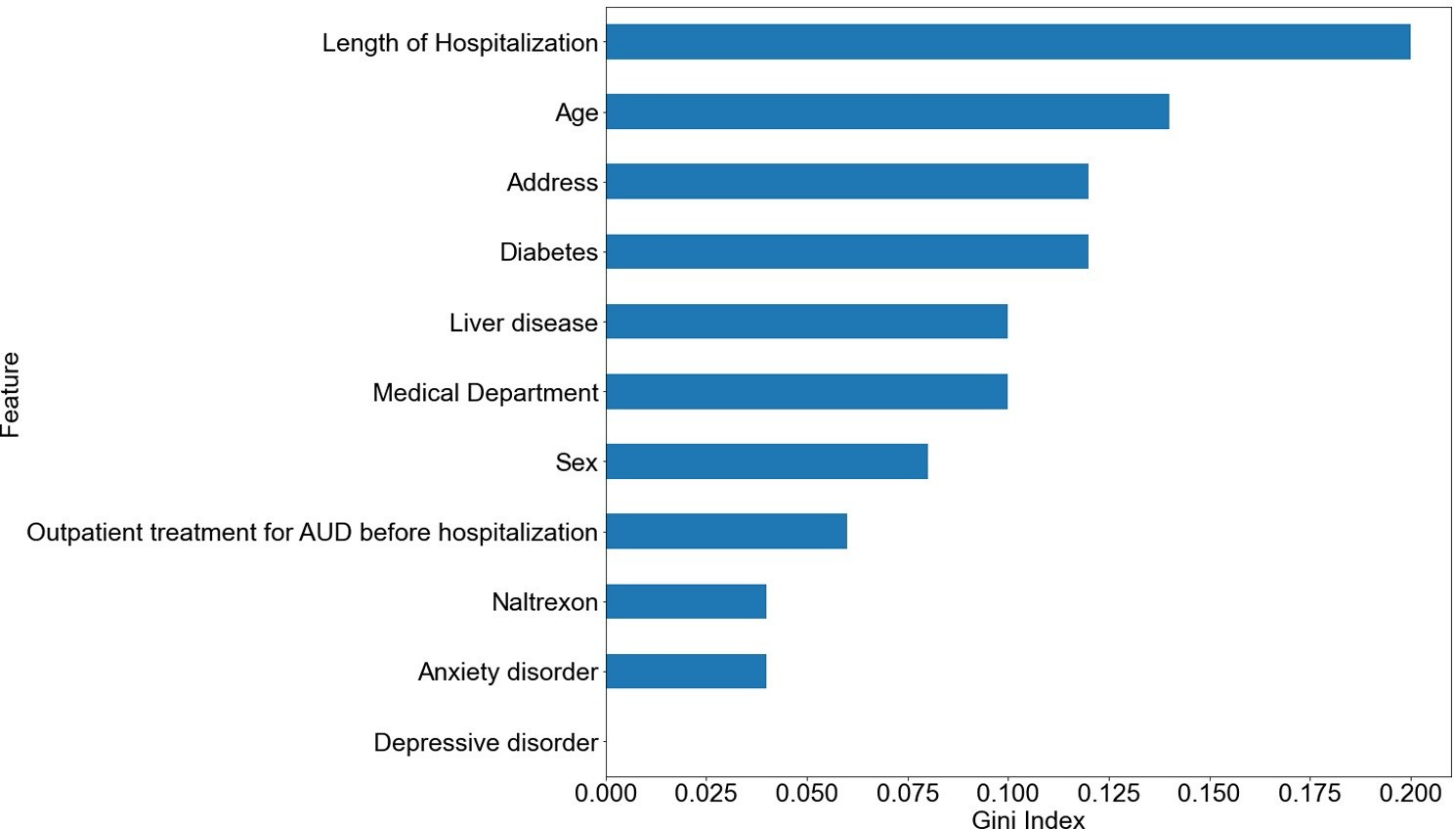
Feature importance of AdaBoost decision tree.

Fig 4 shows the feature importance results obtained using the Gini index [41] in the AdaBoost decision tree. The top four variables that affect patients with AUDs dropping out of treatment are: length of hospitalization, age, region and diabetes.

**Fig 5:**
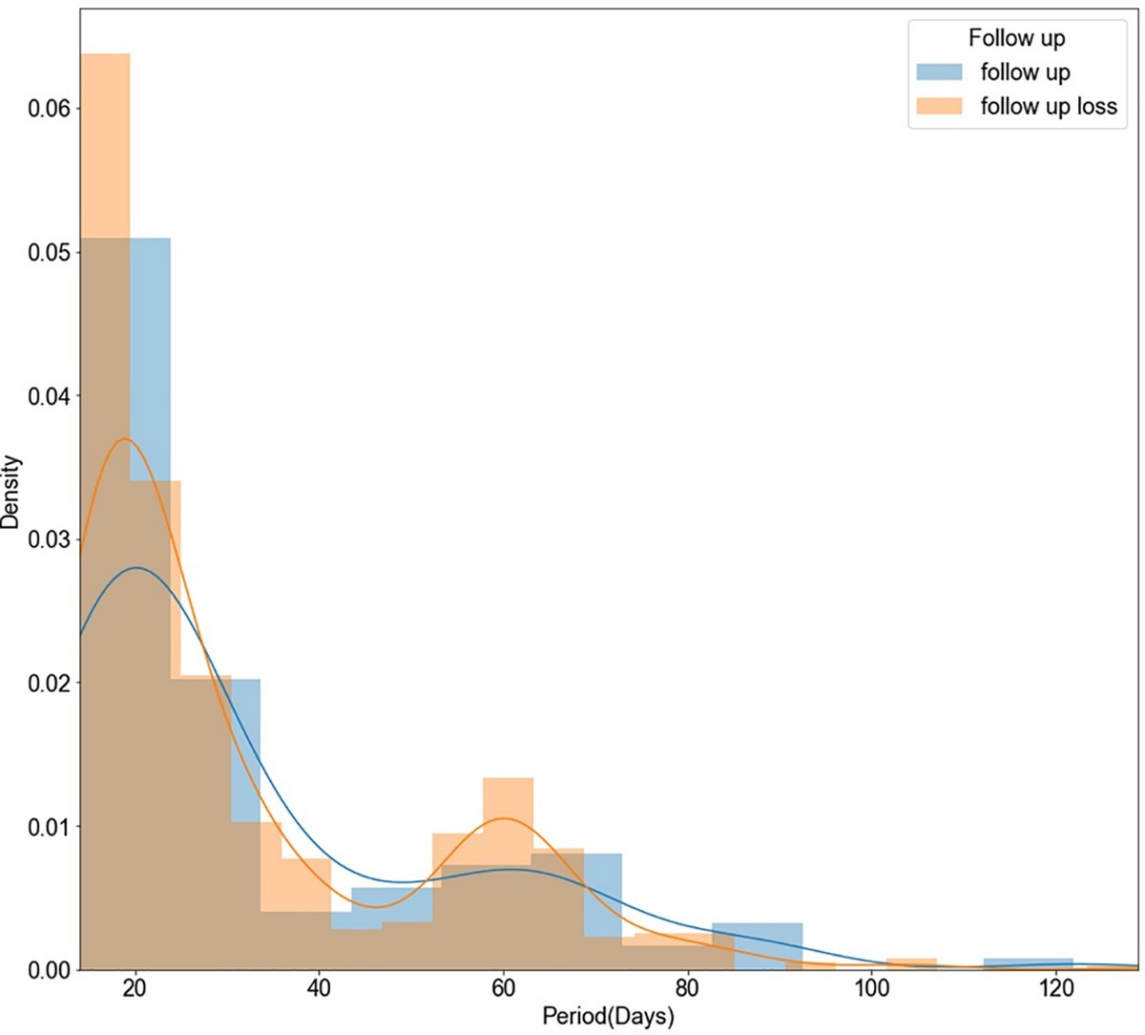
Density plot of length of hospitalization.

According to Fig 5, 61% of all patients were hospitalized within a month. In particular, the proportion of patients who stopped outpatient treatment early was relatively higher than those who did not when the length of hospitalization was 14–25 days.

## Discussion

We developed an algorithm to predict the patients who dropped out of outpatient treatment after discharge from AUD in Korea. This study is relevant as it is the first to use ML. Among the six models, the AdaBoost decision tree had the highest AUROC.

The following are the top four variables affecting discontinuation of outpatient treatment, derived from AdaBoost.

First, the length of hospitalization for AUDs can have an impact on predicting whether outpatient treatment is discontinued. Therefore, special care is needed that patients receive continuous treatment in consideration of the their length of hospitalization. According to the analysis, most of the patients who stopped treatment were hospitalized within 25 days. Based on this information, special care is needed to patients within 25 days of hospitalization. Second, the age at the time of hospitalization for AUD was related to whether treatment was discontinued after discharge. This result was consistent with existing studies that showed increased motivation for continuous treatment with age [42]. Management measures will be needed to increase the willingness of patients in their 40-50s to continue treatment.

Third, the residential area acted as a variable affecting the discontinuation of outpatient treatment. Prior studies also indicate that the higher the accessibility to residential areas and hospitals, the higher the possibility of continuous treatment [43]. As residents outside Seoul and Gyeonggi Province have limited access to hospitals, a system to monitor them in connection with primary institutions will be needed.

Fourth, the presence of diabetes affects the compliance of outpatient treatment. According to the data, the proportion of patients with diabetes in the follow-up group was higher than that in the follow-up loss group. Excessive alcohol consumption by diabetic patients can worsen blood sugar control, which can be fatal to diabetes treatment and, if severe, can lead to death [44]. Diabetes is a chronic disease; therefore, continuous management is needed. However, drinking and diabetes self-management performance are negatively correlated [45]. Patients with diabetes feel the need for alcohol treatment in terms of diabetes management. Moreover, it is estimated that they are more willing and demanding of outpatient treatment. Therefore, continuous care should be maintained in patients without diabetes than in patients with diabetes.

This study however has several limitations. First, there is a problem with inaccuracy regarding the information on patients’ comorbidities. Due to the nature of the retrospective study, the patient’s disease can be identified by the diagnostic code patients received at the hospital where they received treatment. Therefore, it may be challenging to identify all undiagnosed comorbidities in hospitals. However, this study addressed the limitations by using medical records in the hospital from one year before hospitalization.

Second, the data for the study did not include socioeconomic factors and variables related to addiction treatment. Socioeconomic factors such as the patient’s religion, marital status, and occupation, as well as the degree of alcoholism and treatment process, are known to affect the continuous outpatient treatment of patients with AUD [46–48]. However, the CDW that collected the data was then not collecting this information or was under construction. Future studies on predictive models that consider various factors will further reflect clinical reality.

This is the first study in Korea to develop an algorithm that predicts whether patients with AUD will drop out of treatment using ML methods. The final selected AdaBoost algorithm showed higher accuracy than that of traditional models, such as regression analysis. The algorithm could identify key variables affecting treatment discontinuation. This model can be used to develop a clinical decision support system. In other words, this study allows clinicians to assist patients with AUDs in receiving continuous treatment. Moreover, this ML model predicts discontinuation of outpatient treatment in patients with AUD and identifies its factors, but also has the potential to be applicable to other substances.

Finally, our data for analysis included only structured data, and did not include unstructured data elements such as clinical notes. In future studies, including both unstructured and structured data may further improve prediction accuracy.
